# Practical Notes and Formulæ

**Published:** 1877-08-20

**Authors:** 


### Practical Notes and Formulæ.

THOMPSON'S EYE WATER.

R.—Sulphate of copper.........10 grs.

Sulphate of zinc............40 "

Rose water.................. 2 pints.

Tinc. saffron.................

Tinc. camphor aa.......... 4 drs.

Mix and filter.

TOOTHACHE DROPS.—It is convenient for the practitioner to keep on hand a preparation for toothache. The following we clip from *Druggist Circular:*

I. Chloral hydrate............20 grains.
Camphor....................15 "
Chloroform.................30 minims.
Tincture of aconite root..... 5 drops.
Oil of cloves...............10 "
Tincture of opium..........20 "

II. Sulphuric ether ............ 7 fl. drachms.
Chloroform.................. 7 "
Oil of cloves............... 2 "
Camphor.................... 3 drachms.

III. Carbolic acid.............. 1 drachm.
Hydrate of chloral.......... 2 drachms.
Tincture of aconite.........30 minims.
Tincture of opium.......... 4 drachms.
Oil of peppermint........... 1 drachm.

INFANTILE COLIC.—Dr. J. P. F. Brunner, of Topton, Pa., writes to *Journal Mat. Med.:* "I frequently have been called to treat cases of infantile colic, and found the use of opiates to produce only a temporary relief, and finally stun the system. I therefore present the profession the following prescription, which I find gives almost instantaneous relief, and effects a permanent cure:

R—Tinct. asafœtidæ...............gtt. xv.
Tinct. cinnamomi.............f. oz. ss.
Sodii Carbonas...............dr. i.
Syrup, Rhei Aromat.........f. drs. iii.
Aquæ Fontanæ...............f. oz. iss.
M.—Dose, half a teaspoonful ever three hours."

TONIC DOSES OF MERCURY.—Dr. E. L. Keys, New York, in a paper published in the *American Journal Medical Sciences*, January, 1876, gave the results of experiments with minute doses of mercury in the treatment of syphilis, or as he styles them, *tonic* doses of mercury. He begins with the protiodide, gradually increased until impending ptyalism, etc. The dose, at which this symptom occurs, is called the "full dose." This, with opium added, if necessary, is continued until the syphilitic symptoms disappear. The full dose is then reduced one-half, and constitutes the *tonic dose.* This he continues until some fresh symptom of the disease appears, when the half of the full dose, which was dropped, is again added, continued and dropped as before. He follows up this for a period of two to three years, as the only sure method of eradicating the disease.

A USEFUL PASTE.—To every two tablespoonsful of the best wheaten flour add a teaspoonful of common moist or brown sugar, and a little corrosive sublimate; the whole to be boiled, and continually stirred to prevent getting lumpy, till of the right thickness. To stop mouldiness, add a few drops of some essential oil, as lavender or peppermint. This paste is used to make different thicknesses of card board for architectural and similar models. In putting or jointing these together, use the following: to six ounces of gum-arabic (best) add one ounce, or less, of moist or lump sugar, one teaspoonful of lavender or other essential oil, and a tablespoonful of gin, the whole to be mixed in cold water to the consistency of a thick syrup, no heat being in any way applied. This paste can be used for fastening paper firmly to tin.—*Journal Chemistry.*

DIET FOR INFANTS.—In Dunglison's late Reference Book we are told if the milk should disagree, a little lime water should be added, say a tablespoonful to a pint. If pure milk cannot be got, try the condensed milk, which more frequently agrees with the child. It is prepared by adding one tablespoonful of the milk to ten of boiling water, or more, if the child be older. No sugar is required. If this disagrees, try pure cream, diluted with three-fourths of water. By all means keep the nursing-bottle perfectly clean by frequent scalding and washing.

SEDATIVE ANTISEPTIC.—

℞. Chloral hydrate..............

Salicylic acid................

Glycerine.....................

Sulphite soda...............each $1\frac{1}{2}$ parts

Distilled water...............$3\frac{1}{2}$ parts

Spirits of wine...............1 part.

Expose to 100° to 120° (Fahr.) of heat for a few minutes, until the sulphite, salicylic acid and chloral are evolved. Filter through bibulous paper. It can be used both externally and internally. Has been found useful in diptheria, and as an antiseptic generally.

FLAXSEED TEA.—Pour a pint of boiling water over an ounce of whole flax-seed; cover lightly; digest for three or four hours near a fire, and strain; flavor with lemon. A little bruised liquorice root may be added in the preparation; or, if this be disagreeable, may sweeten with sugar, if the patient desire it. Excellent demulcent drink in renal irritations, and to allay cough in pulmonary affections.

COLD MILK PUNCH.—Take a wine-glassful of good brandy, two tablespoonful of water, a tablespoonful of loaf sugar, and a half wine-glassful of rum; add some small lumps of ice; stir together, and add a teacupful of fresh milk, and flavor with nutmeg. In the absence of ice, cold, fresh well or spring water will suffice.

URATES.—These appear only when the urine is cold, and disappear if the urine be heated. They form a heavy precipitate at the bottom of the vessel, with an ill-defined upper border, and are white or deeply tinted by the coloring matter of the urine. They have been called "lateritious deposit," "brick dust deposit," etc.

OXALATE OF LIME.—Does not appear as a distinct sediment, but exists as isolated crystals entangled in a mucus cloud, of octohedral or dumb-bell shape.

URATE OF LIME is a white amorphous powder, of rare occurrence.

SLIPPERY ELM JELLY AND TEA.—Stir four tablespoonsful of bark into a quart of cold water; let it stand all night; in the morning strain and add the juice of one lemon; simmer *gently* twenty minutes,then sweeten and pour into a mold to cool and harden." [*Dunglison.*] Useful as a diet in bowel affections, etc. The tea is made by pouring a pint of boiling water to an ounce of the bark; cover and let stand near the fire for three hours, and strain.

WINE WHEY.—Take fresh milk, a pint, heat to boiling point, and pour in half a pint of white wine; heat to a boil again, being careful not to stir it; as soon as it boils set aside until the curd settles, then pour off the clean whey. If too strong, weaken with water. May be sweetened to suit the taste. It is gently stimulating and nourishing, and well suited to very weak or low conditions of the system.

CARB. AMMONIA IN SNAKE BITE.—Dr. Knott, in *Medical and Surgical Reporter*, reports a case of bite by a large rattlesnake successfully treated with the following solution, repeatedly and freely used, injected with the hypodermic syringe into the veins, half drachm at a time:

℞—Carb. ammonia.......................gr. xl.
Aquæ distil.............................oz. i.
M.—

DISINFECTANT FOR PRIVIES.—The best disinfectant for privies, cesspools, sewers, etc., is a solution of eight pounds of coperas in five gallons of water. If carbolic acid be added it will be still better.

Dry earth has also been found very useful in destroying the offensive odor of privy vaults.

NITRIC ACID TEST FOR ALBUMEN.—If the urine is albuminous slightly, nitric acid added will show a turbid whiteness; if abundant, there will be coagulation, unless soluble nitrate of albumen is formed. If uric acid be in excess, the nitrate of urea will be precipitated, and may be seen with a microscope.

DURATION OF PREGNANCY.—Count 280 days from the beginning of the last menstrual period, and you will not miss it exceeding four or five days, one way or the other.

FOR RINGWORM.—Apply liquor ferri per chloride fortior, mixed with an equal quantity of glycerine, on three successive days. After waiting a few days, reapply if the cure is not complete.

FOR HAEMATURIA.—

℞. Acidi gallici........................... dr. j.
Glycerine................................ oz. j.
Aq. bull.................................. oz. v.

Mix. S. Dose, a tablespoonful.

DIPTHERIA—Citric acid, used locally as spray, or with brush, slightly diluted, has been useful in this disease.

BROWN DISCOLORATION OF SKIN.—

℞. Hydrarg bichlor................. grs. viij.
Boracis pulv..................... dr. ij.
Acid. acet. dil.................... dr. ij.
Alcohol............................. oz. ij.
Aqua, ad............................ oz. iv.
M.
S—Apply to the skin.

URINARARY DEPOSITS (*Dnnglison*) *uric acid.*—Yellow, reddish or brown sediment; little masses of crystals, assuming various forms.

SEBACEOUS GLANDS can be destroyed by the injection of tr. iodine into the sacs through the hypodermic syringe.

BROMIDE OF IRON has been successfully used in incontinence of urine in children; also in hysteria and spermaorrhœa.

FOR ACNE.—

℞. Adipis.................................. dr. v.

Sulphuris...

Acid tinnici. } a a grs. viii. to xv. Mix.

Also, in the morning, bath freely with warm water and a little bay rum, gradually increase the bay rum until one-third of the quantity of rum to water is used.

℞. Zinci cyanidi.................... grs. 1-12.

Pulv. accaciae.

Sach. lactis. } a a........ q. s. Mix.

ft. pill one.

S—Ten of these pil's may be given in twenty-four hours.

CHRONIC DIARRHŒA.—

R. Oxide zinc......... ... ......... 100 gr.

Sodii bicarb....................... 16 "

Div. into chart.................. No. 6.

S—One every three hours.

Salicylate of soda has been used successfully in sciatica, tic, intercostal neuralgia, and in pain of gout, given in seven grain doses, in capsules.

Saturated solution of common salt has been found to relieve the pain of burns, also, common soda.

Epilepsy has been relieved by santonine, in doses from one to five grains, daily.

A few drops of nitric acid, in a glass of sweetened water, will relieve hoarseness.

PHOSPHATES.—In acid urine they appear as a cloudy precipitate, at once soluble, in a drop of nitric acid.

CATARRH SNUFF.—

R. Bismuthe, trisnit................. dr. vi.
Pul. accaciae...... .......... dr. ij.
Morphia muriatis.............. gr. ij.
M.
S—Use occasionally as snuff.

CARBOLIC COLLODIN.—

R—Collodii.........................aa dr. i.
Olei ricini
Acidi carbolic............ . ... oz. i.
M.
Usefcl in chronic ulcers.

SORE NIPPLES.—

R.—Acidi tannici..................... dr. i.
Collodii.......... ............... oz. i.
M.

TOOTHACHE DROPS.—

R.—Creasote
Chloroform... ................. aa dr. i.
Vini opii................. dr. ij.
Tinc. binzoini................ dr. ss.
M.

SALIVATION.—

R.—Acidi Tannici.................. grs. xxx.

Glycerina........ .......... oz. i.

Aquæ...................... oz. vi. M.

S.

Mouth wash.

The Laetate of soda is recommended in cases of insomnia of debilitating diseases, after convalescence, or after hemorrhages.

URATE OF AMMONIA in the utine appears in the form of brown round balls, covered with spines.

OBSTINATE VOMITING—Oxalate of cerum in four grain doses, every two or four hours.

